# The Role of Interventional Radiology in the Treatment of Arterial Diabetic Foot Disease

**DOI:** 10.1007/s00270-016-1337-y

**Published:** 2016-07-19

**Authors:** Jim A. Reekers

**Affiliations:** Department of Radiology, G1.206, Academic Medical Center, University of Amsterdam, Meibergdreef 9, PO Box 22660, 1100 DD Amsterdam, The Netherlands

## Introduction

The role of IR in treatment of diabetic foot disease and Peripheral Arterial Disease (PAD) has been growing very rapidly. It is now widely recognized that endovascular treatment can play a crucial role in prevention of amputation in diabetic patients. It is important that this role is also recognized by those who are the primary caretakers of patients with diabetes. Internationally, the International Working Group on the Diabetic Foot (IWGDF) plays an important role in establishing the evidence for endovascular treatment. The IWGDF, which was founded in 1996, is a non-profit organization with independent funding. The aim of the IWGDF is to create awareness of the disease and to improve the management and prevention of the diabetic foot. One of the main tasks of the IWGDF is to create evidence-based guidelines on the diagnosis, prognosis, and management of peripheral artery disease in patients with foot ulcers [1]. These guidelines are updated every 4 years. In 2015, there was a new update on PAD (www.iwgdf.org).

For those interventional radiologists who want to be involved in the treatment of diabetes and PAD, it is very important to be aware of these guidelines and, moreover, to be part of the local clinical program implementing these guidelines. Diagnosis, prognosis, and management are very closely related and should all be part of IR knowledge, as it is not possible anymore to only perform the endovascular part as a stand-alone intervention. It is of course the technical part of the intervention that will be the specific role of the interventionist, but being part of a multidisciplinary team is mandatory for the best outcome. The evidence-based systemic reviews, as performed by the IWGDF every 4 years, result in a list of recommendations which are approved at the general assembly at the international meeting which is also held every 4 years. This year the meeting was in The Hague. The recommendations are accompanied by 2 remarks, based on the GRADE scale classification and the quality of the evidence for this recommendation. The recommendations are divided over 3 domains: diagnosis, prognosis, and treatment.

## Diagnosis

1. Examine a patient with diabetes annually for the presence of peripheral artery disease (PAD); this should include, at a minimum, taking a history and palpating foot pulses (GRADE recommendation: strong; quality of evidence: low).
2. Evaluate a patient with diabetes and a foot ulcer for the presence of PAD. Determine, as part of this examination, ankle or pedal Doppler arterial waveforms; measure both ankle systolic pressure and systolic ankle brachial index (ABI) (Strong; Low).
3. We recommend the use of bedside non-invasive tests to exclude PAD. No single modality has been shown to be optimal. Measuring ABI (with <0.9 considered as abnormal) is useful for the detection of PAD. Tests that largely exclude PAD are the presence of ABI > 0.9–1.3, toe brachial index (TBI) >0.75, and the presence of a triphasic pedal Doppler arterial wave forms (Strong; Low).

## Prognosis

4. In patients with a foot ulcer in diabetes and PAD, no specific symptoms or signs of PAD reliably predict healing of the ulcer. However, one of the following simple bedside tests should be used to inform the patient and healthcare professional about the healing potential of the ulcer. Any of the following findings increases the pre-test probability of healing by at least 25 %: a skin perfusion pressure ≥40 mmHg; a toe pressure ≥30 mmHg; or a TcPO2 ≥25 mmHg (Strong; Moderate).
5. Consider urgent vascular imaging and revascularization in patients with a foot ulcer in diabetes where the toe pressure is <30 mmHg or the TcPO2 <25 mmHG (Strong; Low).
6. Consider vascular imaging and revascularization in all patients with a foot ulcer in diabetes and PAD, irrespective of the results of bedside tests, when the ulcer does not improve within 6 weeks despite optimal management (Strong; Low).
7. Diabetic microangiopathy should not be considered to be the cause of poor wound healing in patients with a foot ulcer (Strong; Low).
8. In patients with a non-healing ulcer with either an ankle pressure <50 mmHG or ABI < 0.50, consider urgent vascular imaging and revascularization (Strong; Moderate).

Ad 4–6. It is important to discuss these criteria in the multidisciplinary team. It is known that these parameters for treatment can be very unreliable and often a diabetic foot can be at risk at much higher ABI, toe pressure, or TcPO2 [2, 3]. The clinical situation should always prevail over these measurements. Being part of a multidisciplinary team will also in this situation be a condition sine qua non.

## Treatment

9. Color Doppler ultrasound, CT angiography, MR angiography, or intra-arterial digital subtraction angiography can each be used to obtain anatomical information when revascularization is being considered. The entire lower extremity arterial circulation should be evaluated, with detailed visualization of below-the-knee and pedal arteries (Strong; Low).
10. The aim of revascularization is to restore direct flow to at least one of the foot arteries, preferably the artery that supplies the anatomical region of the wound, with the aim of achieving a minimum skin perfusion pressure ≥40 mmHg; a toe pressure ≥30 mmHg; or a TcPO2 ≥25 mmHg (Strong; Low).
11. A center treating patients with a foot ulcer in diabetes should have the expertise in and rapid access to facilities necessary to diagnose and treat PAD; both endovascular techniques and bypass surgery should be available (Strong; Low).
12. There is inadequate evidence to establish which revascularization technique (endo or bypass) is superior and decisions should be made in a multidisciplinary team on a number of individual factors, such as morphological distribution of PAD, availability of autogenous vein, patient co-morbidities, and local expertise (Strong; Low).
13. After a revascularization procedure for a foot ulcer in diabetes, the patient should be treated by a multidisciplinary team as part of a comprehensive care plan (Strong; Low).
14. Patients with signs of PAD and a foot infection are at particularly high risk for major limb amputation and require emergency treatment (Strong; Moderate).
15. Avoid revascularization in patients in whom, from the patient perspective, the risk–benefit ratio for the probability of success is unfavorable (Strong; Low).

Ad. 9 The role of the interventional radiologist is to advise on further diagnostic imaging in case PAD is suspected and there is the clinical need for treatment.

If further diagnostic imaging is needed, the most cost-effective way is to perform Color Doppler ultrasound imaging. This technique is very reliable but also operator dependent [4, 5]. Alternatively both CTA and MRA can be advised. A recent systemic review and meta-analysis showed that both computed tomography and contrast-enhanced magnetic resonance angiography can evaluate arteries in peripheral arterial disease. CTA and CE-MRA have been shown to be accurate. Regarding the diagnostic performances in critical limb ischemia they are not different. CTA and CE-MRA can distinguish confidently between high-grade stenoses and occlusions. Often, a separate imaging technique of tibial arteries by CE-MRA is preferred [6]. The alternative way to extend the diagnosis is by combining diagnosis and treatment in one session by direct use of angiography. With a good clinical team and good work-up, the latter is certainly an important option to consider.

Ad 10. Although the evidence for the angiosome theory is confusing and not very strong, the guideline suggests that, if possible, the vessel to the angiosome or direct revascularization should be the first choice, especially in diabetic patients [7, 8].

Ad 11–12. Neither bypass nor endovascular surgery can claim superiority in outcome based on the current evidence. In the 2011 IWGDF guideline it read that there is not enough evidence to make a choice between bypass surgery and endovascular treatment. Here again the multidisciplinary team plays a crucial role. Topics for discussion in the team are morphological distribution of PAD, availability of autogenous vein, patient co-morbidities, and local expertise. For the interventional radiologists, expertise and maintaining expertise is a very important factor. The latter can only be obtained if every IR group has only a limited number of clinically dedicated and trained IRs for the treatment of diabetic foot disease. Local and international training programs and accreditation- and outcome-based assessments can increase the level of expertise and IR quality.

### New Endovascular Technologies

Although the new IWGDF guideline and recommendations do not state anything about new endovascular technologies, from an IR perspective these innovations are very important. Unfortunately, many of the current (case) series describing the results of new drug-eluting technologies, like drug-eluting balloons, and drug-coated stents are of poor scientific quality. Many are at high risk of bias with confounding by indication and no attempts being made to adequately adjust for ulcer duration or severity of disease. Many studies, often performed with full control of the sponsoring pharmaceutical company, are dominating the data pool for a systematic review. A recent systematic review comparing all these new technologies could only show some small benefits for proxy endpoints like patency, TLR, and binary restenosis, but no clinical benefit regarding wound healing or prevention of amputation [9]. All of the published data on these new technologies also come from patient groups where diabetes and atherosclerosis are mixed. Currently, simple PTA with optional bailout stenting still remains the technique of choice. High-quality randomized studies are very much needed here.

## In Conclusion

Interventional radiology plays a crucial role in the treatment of diabetic foot disease. It is however essential for any IR in this field to be fully clinically dedicated and to be part of a multidisciplinary team. Also obtaining and maintaining personal expertise is very important. The latter could be expressed in national centers of excellence with well-defined criteria. It is important to stay closely connected to new endovascular developments but never to forget the principle of evidence-based medicine. It is to be expected that diabetic foot disease, and the cost related, will further increase, so preparing for the future with dedicated training programs and manpower is important.
